# The Clinical Application of Robot-Assisted Ventriculoperitoneal Shunting in the Treatment of Hydrocephalus

**DOI:** 10.3389/fnins.2021.685142

**Published:** 2021-08-05

**Authors:** De-feng Liu, Huan-guang Liu, Kai Zhang, Fan-gang Meng, An-chao Yang, Jian-guo Zhang

**Affiliations:** ^1^Department of Neurosurgery, Beijing Tiantan Hospital, Capital Medical University, Beijing, China; ^2^Department of Functional Neurosurgery, Beijing Neurosurgical Institute, Capital Medical University, Beijing, China; ^3^Beijing Key Laboratory of Neurostimulation, Beijing, China

**Keywords:** ventriculoperitoneal shunting, robot-assisted implantation, effectiveness, safety, hydrocephalus

## Abstract

**Background:**

This work aims to assess the effectiveness and safety of robotic assistance in ventriculoperitoneal shunting and to compare the results with data from traditional surgery.

**Methods:**

We retrospectively analyzed 60 patients who had undergone ventriculoperitoneal shunting, of which shunts were implanted using a robot in 20 patients and using traditional surgical methods in the other 40 patients. Data related to surgery were compared between the two groups, and the accuracy of the drainage tube in the robot-assisted group was assessed.

**Results:**

In the robot-assisted surgery group, the operation duration was 29.75 ± 6.38 min, intraoperative blood loss was 10.0 ± 3.98 ml, the success rate of a single puncture was 100%, and the bone hole diameter was 4.0 ± 0.3 mm. On the other hand, the operation duration was 48.63 ± 6.60 min, intraoperative blood loss was 22.25 ± 4.52 ml, the success rate of a single puncture was 77.5%, and the bone hole diameter was 11.0 ± 0.2 mm in the traditional surgery group. The above are statistically different between the two groups (*P* < 0.05). Only one case of surgery-related complications occurred in the robot-assisted group, while 13 cases occurred in the traditional surgery group. There was no significant difference in the hospitalization time. In the robot-assisted surgery group, the average radial error was 2.4 ± 1.5 mm and the average axial error was 1.9 ± 2.1 mm.

**Conclusion:**

In summary, robot-assisted implantation is accurate, simple to operate, and practical; the duration of surgery is short; trauma to the patient is reduced; and fewer postoperative complications related to surgery are reported.

## Introduction

Hydrocephalus is common in all types of craniocerebral trauma and in the case of an intracranial mass, which leads to the progressive dilatation of the ventricular system and/or subarachnoid space due to the disturbance of absorption, circulation, or excessive secretion of cerebrospinal fluid. Surgical treatments include insertion of a ventriculoperitoneal shunt (VPS) and endoscopic third ventriculostomy (ETV), of which VPS is the most commonly used method for the treatment of all types of clinical hydrocephalus. VPS involves the insertion of a ventricle-end drainage tube into the ventricle through a skull drill. Several commonly used lateral ventricle puncture methods are lateral ventricle frontal puncture, traditional occipital puncture, and triangle puncture. The drainage tube is connected to a shunt valve (to control the flow rate of cerebrospinal fluid), then the abdominal cavity-end drainage tube is placed under the skin into the abdominal cavity through a tunnel. Traditional surgery requires marking the body surface anatomy of the patient and determining the trajectory of the drainage tube into the skull based on these marks. The precise placement of the drainage tube in traditional surgery is vital, but it is difficult to control the position and length of the drainage tube in the ventricle due to the different sizes of the ventricles of each patient. Various complications such as incorrect placement, infection, bleeding, and obstruction of the shunt system may occur after traditional VPS surgery, which leads to poor surgical results.

In neurosurgery, there are often space-constrained, high-precision, intensive, and tedious tasks, and the emergence of robots has the potential to simplify procedures and improve accuracy (Guo et al., 2018). Neurosurgery robots overcome the problems of poor accuracy, long operation times and fatigue of the surgeon, and lack of 3D precise vision in traditional surgical operations.

The Remebot robotic system is a frameless stereotactic product and neurosurgery assistance tool. The robot includes a computer software system, a six-axis robotic arm, and a camera. The surgeon can use the computer software system to observe multimodal images of the head and plan the best surgical puncture path. The arm can help the surgeon accurately locate the puncture site for the operation, and can act as a multifunctional operation platform. The camera can perform spatial mapping and real-time tracking and ensure that the robotic arm moves along the planned path to the preoperative planned position. The videometric tracker integrated by the Remebot robotic system is a commercially available third-generation stereoscopic optical tracking product. The product is fully passive and uses available visible light illumination to detect and track objects of interest, much as humans do, by triangulating 3D poses between two video cameras with overlapping projections (Choi et al., 2019).

This device is intended for the spatial positioning and orientation of neurosurgical instruments and is potentially applicable to any neurosurgical condition in which the use of stereotactic surgery may be appropriate, such as the implantation of DBS electrodes, the implantation of intracerebral electrodes for SEEG, biopsies of intracerebral lesions, puncturing of cysts, and evacuation of hemorrhages, as well as navigation for open neurosurgeries. For example, in deep brain stimulation surgery (von Langsdorff et al., 2015; Goia et al., 2019; VanSickle et al., 2019), the accuracy, safety, and stability of robot-assisted electrode implantation have been proven.

Based on compelling evidence of their accuracy, steadiness, and endurance, robotic systems are promising for use in drainage tube placement. In other words, robot assistance could help to place the shunt tube in the optimal position in the ventricle, thereby reducing the incidence of postoperative complications.

In this study, 60 patients who underwent ventriculoperitoneal shunting surgery in Beijing Tiantan Hospital from June 2018 to September 2020 were selected, of which shunts were implanted with robot assistance in 20 patients and by traditional surgical methods in 40 patients. After surgery, the precise position of the intracranial shunt, the depth of implantation, and surgical trauma were analyzed.

## Materials and Methods

### General Data

Sixty patients who underwent ventriculoperitoneal shunting surgery in Beijing Tiantan Hospital from June 2018 to September 2020 were selected, 20 of which had shunts implanted with robot assistance and 40 received shunts *via* traditional surgical methods. The average age of group A was 24 ± 19.59 years, and this group comprised 11 males and 9 females; the average age of group B was 30.65 ± 19.46 years, and this group comprised 22 males and 18 females. There was no significant difference in age or sex between the two groups of surgical patients. The patients and their family members voluntarily chose the operation method before surgery. All the patients in this study provided informed consent and signed the operation informed consent form. This study was approved by the Ethics Committee of Beijing Tiantan Hospital (Grant No. QX201600-706).

### Surgical Procedure

#### Robot-Assisted Ventriculoperitoneal Shunting

##### Preoperative Planning and the Operative Procedure

All patients underwent magnetic resonance imaging (MRI) (3.0 Tesla, Siemens, Munich, Germany) before surgery. To guarantee the visualization of the anatomical structures of interest, sagittal and axial volumetric T1-weighted MRI (slice thickness 1.0 mm, TR 6.4 ms, TE 3.0 ms, interslice gap 0 mm, flip angle 8°) were performed. The images were then compiled to plan the targets and trajectories.

**FIGURE 1:**
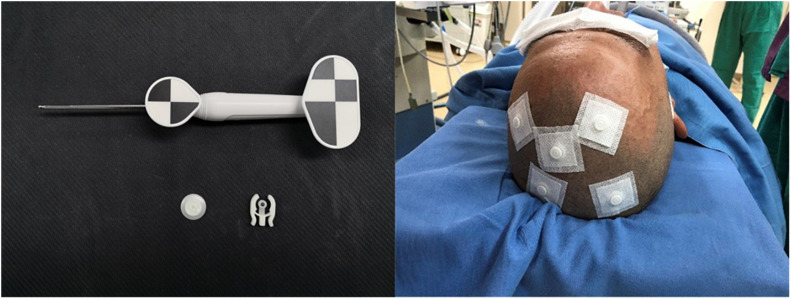
Videometric-tracked marker: it was capable of automatic patient-to-image registration, was adhered to the scalp in the preoperative and accompanied the patient for a CT scan; detected optical frame marker during surgery.

**FIGURE 2:**
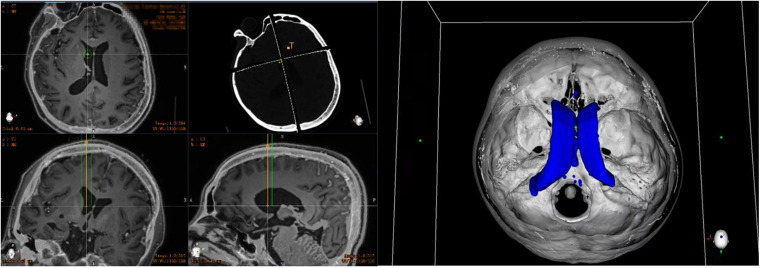
Preoperative planning of robotic work platform: automatically calculate the 3D ventricle segmentation of the patient and plan the target and cranial path before surgery (the orange line represents the implanted drainage tube, the green line represents the safe distance for the drainage tube implantation, and the blue shape represents the ventricle of the patient).

On the day of surgery, a dedicated videometric-tracked marker, referred to as the optical frame marker, which was capable of automatic patient-to-image registration, was adhered to the scalp, avoiding injury (Figure 1). Following this, axial volumetric computed tomography (CT) (slice thickness 0.625 mm, interslice gap 0 mm, 120 kVp) was performed. All images were loaded into the Remebot software, and MR images were fused to the CT images as the reference examination due to MRI distortions (Benabid et al., 2009; Guo et al., 2018). After segmenting the 3D objects of interest, surgical planning was performed. The robot working system can automatically calculate the 3D ventricle segmentation of the patient, that is, by selecting the appropriate ventricular region threshold, the ventricular region and other tissues can be distinguished by the image gray level and the pixel level can be divided. Thereby, the ventricular region and some cerebrospinal fluid can be segmented. Then, the area, shape, and other characteristics of the connected domain are used to distinguish and exclude non-ventricular parts, obtaining a high-precision ventricle segmentation result. The trajectory required to reach that location was planned on 3D objects or any available view, avoiding vessels and nerves (Figure 2).

**FIGURE 3:**
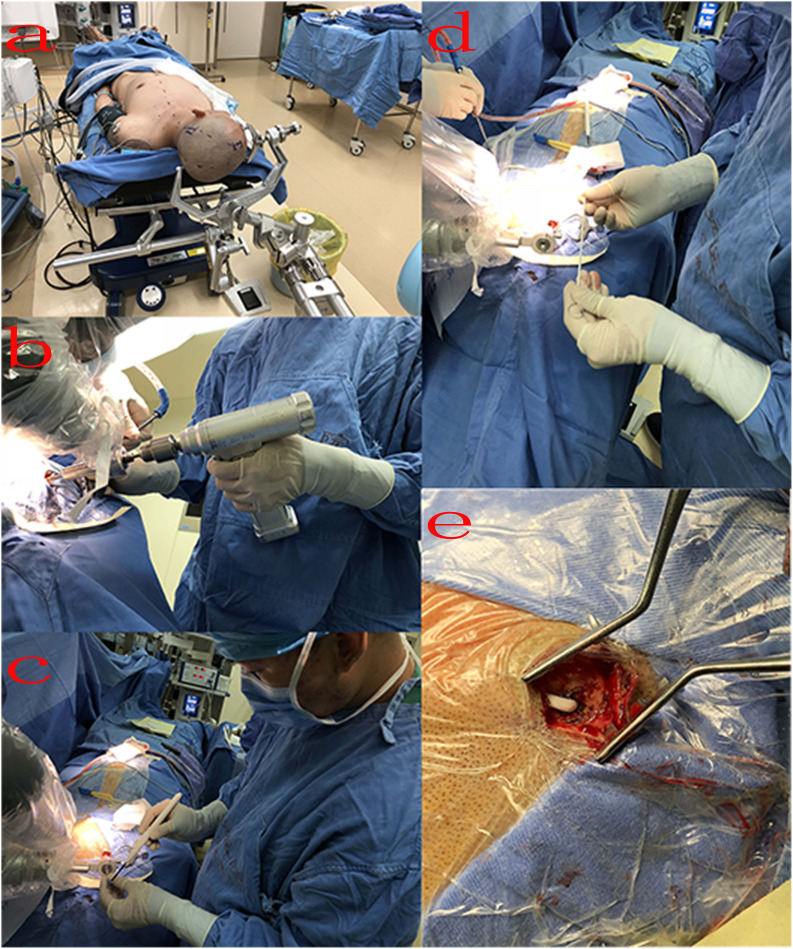
Intraoperative process: **(A)** surgical position: the patient was placed in the supine position, and the Mayfield headholder was positioned and secured to the trolley with a mechanical support arm; **(B)** surgeon drills a bone hole; **(C)** penetrate the dura using a monopolar electric knife; **(D)** implant drainage tube; **(E)** after implantation, the drainage tube was placed under the skin.

In the operating room, the patient was placed in the supine position and the Mayfield headholder was positioned to avoid any interference. The mobile trolley was stabilized on the left side of the patient and the Mayfield headholder was secured to the trolley with a mechanical support arm to establish rigid immobilization between the head of the patient and the robotic arm (Figure 3A). The videometric tracker (MicronTracker, ClaroNav, Toronto, Canada), with three stereotactic cameras held by an independent stand, was installed above the head of the patient, where the optical marker could be detected (Figure 1). Next, correlations of the different spaces were carried out, involving two steps, namely, (1) tracker-to-image registration and (2) tracker-to-robot registration. The Remebot robotic system features a paired point-based, automatic registration. At the end of the tracker-to-image registration, the registration error was validated less than 0.3 mm. The tracker-to-robot registration was achieved by correlating two sets of spatial positions from the robotic arm space and the tracker space. A fiducial point was defined on the videometric-tracked target pattern engraved on the end effector attached to the robotic arm. During registration, the robotic arm automatically moved to certain poses surrounding the head of the patient, and the coordinates of that fiducial point in separate spaces were automatically obtained from the robot forward calculation and the tracker. At the end of the tracker-to-robot registration, the registration error was validated as less than 0.08 mm. Subsequently, the robot-to-image registration was accomplished by relying on the above correlations, and data could be transferred between the images and the robotic arm.

Following the registration, the robotic arm was oriented on command to the trajectories, and the scalp entry points were marked. After draping and local anesthesia, scalp incisions and burr-hole drillings were performed under the guidance of the robotic arm, but the dura was not opened to prevent untimely cerebral spinal fluid loss and subsequent brain shift (Figure 3B). The dura mater was penetrated with unipolar electrocautery (Figure 3C). In case of any possible displacement of the head of the patient, the automatic registration was efficiently repeated. Once completed, the accuracy of the registration was visually inspected at less than 0.5 mm by commanding the robotic arm to guide a tooltip of 1 mm in diameter into two holes of 2 mm in diameter on the optical frame marker, according to the preoperative planning. Afterward, the robotic arm moved to a target point and was oriented to the trajectory with a microdrive device. The dura was perforated and cannulas were advanced to the defined depth.

The drainage tube enters the ventricle along the cannula, and the vital signs and symptoms of the patient were constantly observed during the process (Figure 3D). When necessary, the drainage tube was altered through micromovements of the robotic arm by submillimeter steps as small as 0.1 mm. When the physiological and clinical criteria for successful tube placement were fulfilled, the drainage tube was anchored to the skull. The subsequent surgical procedure of placing the drainage tube through the subcutaneous tunnel into the abdominal cavity was similar to the previous surgery (Figure 3E).

**FIGURE 4:**
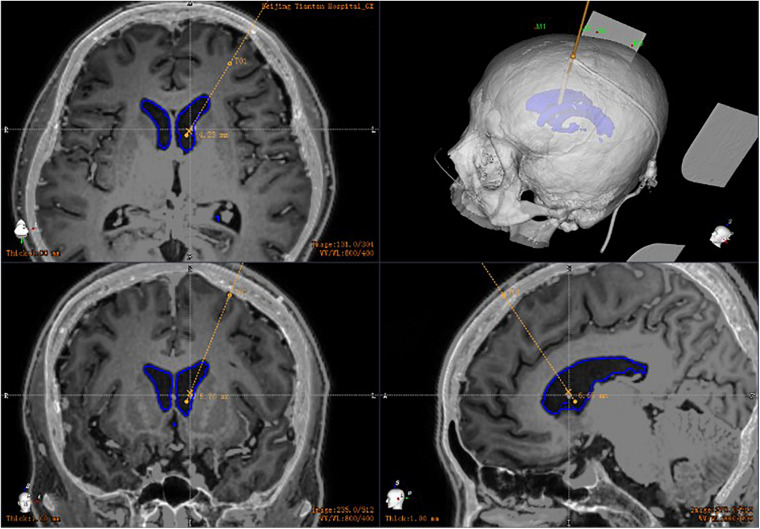
Postoperative drainage tube reconstruction: postoperative image fusion, 3D reconstruction of the position of the intracranial drainage tube; the drainage tube is accurately implanted into the ventricle.

All patients had a postoperative CT scan (slice thickness 0.625 mm, interslice gap 0 mm, 120 kVp). The CT images were matched with the preoperative planning to assess the drainage tube placement accuracy (Figure 4). The drainage tube accuracy was the deviation between the actual center of the implanted tube and the intended target point and was assessed using two types of measurements (Starr et al., 2010; VanSickle et al., 2019): the “radial error,” defined as the scalar distance measured from the view perpendicular to the planned trajectory, and the “axial error,” defined as the scalar distance along the planned trajectory measured from the view along the planned trajectory. The distance between the tip of the ventricle of the drainage tube and the interventricular foramen was calculated. All patients were followed up to verify associated complications, such as hemorrhage, infection, or poor incision healing.

##### Traditional Ventriculoperitoneal Shunting

In the operating room, the patient was placed in the supine position and the Mayfield headholder was positioned. The surgical site was sterilized with iodine and alcohol.

The intersection of 2 cm before the coronal suture or within the hairline and 2.5 cm from the median sagittal line was marked. After local anesthesia, a skull cone or a skull drill was used to drill a hole to reach the dura. A puncture needle was then used to puncture through the drill hole. The puncture direction was parallel to the sagittal plane, and the needle tip was backward and facing down, aligned with the line of the external auditory canal on both sides. After piercing 3–4 cm and a sense of breakthrough, the needle core was pulled out. When the cerebrospinal fluid began to flow, the drainage tube was implanted 1–2 cm deep to ensure its location in the ventricle. The puncture point of the occipital angle puncture was 6 cm above the extraoccipital tuberosity and 2.5–3 cm beside the midline, and the puncture direction pointed to the midpoint of the brow arch on the same side. All patients had a postoperative CT scan (slice thickness 0.625 mm, interslice gap 0 mm, 120 kVp).

### Statistical Analysis

SPSS 23.0 software (IBM SPSS Statistics Inc., Chicago, IL, United States) was used for statistical analysis. The experimental results are expressed as the mean ± standard deviation (*x* ± *s*). The normality and homoschedasticity of the two groups of data were detected. If the variance was aligned, a one-way analysis of variance was performed, and if the variance was not uniform, the Wilcoxon test was performed. *P* < 0.05 was considered statistically significant.

## Results

### Comparison of the Characteristics of Patients in the Robot-Assisted and Traditional Surgery Groups

A total of 60 drainage tubes were implanted in 60 patients. The mean age of the patients in the robot-assisted surgery group was 24 ± 19.59 years (range, 1–66 years), whereas the mean age of the patients in the traditional surgery group was 30.65 ± 19.46 years (range, 1–66 years). In this study, there was no significant difference in age or sex between the two groups (Table 1).

**TABLE 1:** Comparison of the characteristics of patients in the robot-assisted and traditional surgery groups.

Variables	Robot-assisted ventriculoperitoneal shunting (*n* = 20)	Traditional ventriculoperitoneal shunting (*n* = 40)	*P*-value
Age (*M* ± SD)	24 ± 19.59	30.65 ± 19.46	0.218
Gender, *N* (%)			0.584
Male	9 (45%)	21 (52.5%)	
Female	11 (55%)	19 (47.5%)	

### Comparison of Clinical Characteristics in the Robot-Assisted and Traditional Surgery Groups

In the robot-assisted surgery group, the operation duration (from the completion of disinfection and draping to the fixing of the drainage tube) was 29.75 ± 6.38 min, intraoperative blood loss was 10.0 ± 3.98 ml, the successful rate of once puncture was 100%, and the diameter of the bone hole for robot-assisted implant surgery was 4.0 ± 0.3 mm, which all differed statistically from the data obtained from the traditional surgery group (Table 2) (*P* < 0.05). In the robot-assisted surgery group, the average radial error was 2.4 ± 1.5 mm and the average axial error was 1.9 ± 2.1 mm. In the traditional ventriculoperitoneal shunting group, the average operation time was 48.63 ± 6.60 min, the intraoperative blood loss was 22.25 ± 4.52 ml, the successful rate of once puncture was 77.5%, and the average bone hole diameter was 11.0 ± 0.2 mm.

**TABLE 2:** Comparison of clinical characteristics in the robot-assisted and traditional surgery groups.

Variables	Robot-assisted ventriculoperitoneal shunting (*n* = 20)	Traditional ventriculoperitoneal shunting (*n* = 40)	*P*-value
Operation duration	29.75 ± 6.38 min	48.63 ± 6.60 min	4.04034E-15
Intraoperatve blood loss	10.0 ± 3.98 ml	22.25 ± 4.52 ml	1.09E-14
The hospitalization time	3.5 ± 2.2 days	4.2 ± 1.5 days	0.12
Successful rate of once puncture	100% (20/20)	77.5% (31/40)	
Bone hole diameter	4.0 ± 0.3 mm	11.0 ± 0.2 mm	
Surgery-related Complications			
Puncture tract bleeding	1	10	
Infection	0	3	
Contact choroid plexus	0	15	
Accuracy			
Radial error	2.4 ± 1.5 mm	–	–
Axial error	1.9 ± 2.1 mm	–	–

In the robot-assisted surgery group, only one case of surgery-related complications occurred, and a small amount of bleeding from the puncture tract required clinical observation. In addition, the puncture process completely avoided the choroid plexus, and none of the patients contacted the choroid plexus during the drainage tube puncture. In the traditional ventriculoperitoneal shunting group, 13 cases of surgery-related complications occurred, namely, 10 cases of bleeding and 3 cases of infection. In 15 of the 40 patients (37.5%), the drainage tube came into contact with the choroid plexus during the puncture.

## Discussion

Traditional surgical methods are prone to complications such as bleeding, infection, and shunt obstruction. Infection is one of the most serious complications, with an incidence of 4–11% (Kestle et al., 2011; von der Brelie et al., 2012). Infections are mainly related to intraoperative operations. Preventive use of antibiotics, strict aseptic techniques, and delicate and skilled surgical operations can reduce the occurrence of infections. The improper placement of the drainage tube has a high incidence rate, mainly resulting in the tube needing to be removed or reset (Nesvick et al., 2015). According to statistics, in all patients with VPS, about 30% have improper placement of the drainage tube tip, and about 20% require repositioning. Therefore, accurate placement of the tip-end drainage tube is a critical clinical procedure in need of improvement.

Therefore, a fast and accurate ventricular puncture guidance method is urgently needed. Robotic assistance is one promising approach. The robot working system can automatically calculate the 3D ventricle segmentation of the patient, that is, by selecting the appropriate ventricular region threshold, the ventricular region and other tissues can be distinguished by the image gray level and the pixel level can be divided. Thereby, the ventricular region and some cerebrospinal fluid can be segmented. Then, the area, shape, and other characteristics of the connected domain are used to distinguish and exclude non-ventricular parts, obtaining a high-precision ventricle segmentation result. The trajectory required to reach that location was planned on 3D objects or any available view, avoiding vessels and nerves. The clinical data of 60 patients who underwent robot-assisted ventriculoperitoneal shunting in the Department of Neurosurgery of Beijing Tiantan Hospital affiliated to Capital Medical University from June 2018 to September 2020 were analyzed, 20 of which had shunts implanted with robot assistance and 40 had shunts implanted by traditional surgical methods.

First, robot-assisted surgery can accept smaller bone holes. The DGR-I drill (ACRA-CUT, Acton, MA, United States), used in the conventional operation, had a diameter of 11 mm, whereas the specialized bit, used in the robot stereotactic guided assisted operation, had a diameter of only 4 mm, which could directly drill through the scalp and skull; as a result, the orientation was consistent with the preoperative planning direction. The small diameter and precision of the specialized bit not only can reduce the exposure of brain tissue but also improve patient tolerance. Second, smaller incisions and bone holes mean less bleeding. Intraoperatve blood loss in the robot-assisted surgery group was 10.0 ± 3.98 ml, while the traditional surgery group was 22.25 ± 4.52 ml (*P* < 0.01). Third, using a robot, the drainage tube can be implanted into the ventricle using only one attempt, especially in patients with small ventricles. In our set of data, the success rate of a single puncture was 100% and the rate of contact with the choroid plexus was 0%, which means that the use of robots can not only improve the efficiency of surgery but can also reduce the risk of trauma caused by multiple punctures. As the number of puncture operations increases, the risk of complications such as bleeding and infection also increases, with the risk increasing exponentially with each successive puncture. One study reported that the desired target was hit in only 39.9% of cases during the free-hand insertion of an external ventricular drain (Toma et al., 2009). In another review, only almost half (47.9%) of the catheters were placed with the entire tip located in the cerebrospinal fluid (Wan et al., 2011). What is more, fewer puncture times can effectively reduce complications. In our cohort, only one case of surgery-related complications occurred in the robot-assisted group, while 13 cases occurred in the traditional surgery group.

To compare the operation times between the two groups, we calculated the time from the patient entering the operating room to the end of the operation. The robot-assisted group showed a significantly shorter average operation time than the conventional operation group. The surgical robot can automatically locate the path and target after registration, and then the surgeon can drill a hole, electrocoagulation penetrates the dura mater, and a limited length drainage tube is safely implanted. For the traditional surgery group, skin incision and hand drill are required before drilling, then the dura mater is cut, and finally a drainage tube is implanted. There is no doubt that the procedures have been simplified, and the success rate of puncture has been improved, which has significantly reduced the operation time. In addition, there was no significant difference in the average length of hospitalization between the two groups. This also means that robot-assisted surgery will not increase the burden on patients.

In our study, we compared the error between the planned drainage tube tip target and the actual position after surgery. The average radial error was 2.4 ± 1.5 mm, and the average axial error was 1.9 ± 2.1 mm with robot-assisted implantation. There were no cases of passing through the ventricle into the brain parenchyma and no cases of insufficient drainage tube implantation depth. Robot-assisted drainage tube implantation can effectively control the position of the drainage tube. Implantation into the ventricle occurs in one attempt and the implantation depth of the drainage tube can be controlled, effectively avoiding the choroid plexus and reducing the risk of shunt tube obstruction. Precise disposable catheter placement can minimize the risk of complications from intubation through the brain tissue. Inaccurate catheterization not only fails to achieve drainage function but can also cause accidental brain damage, such as damage to the corticospinal tract, basal ganglia, limbic system, optic nerve, optic tract, posterior commissure, and other tissues, causing dysfunction (Shults et al., 1993; Gold et al., 2008; Torrez-Corzo et al., 2009). For traditional techniques, under visual inspection, it is sometimes difficult by manual operation to ensure that the puncture needle does not bend and deviate from the puncture direction due to the incorrect posture of the patient or a difference in the visual angle of the binocular vision of the surgeon; if the puncture is too deep, it may reach the third ventricle or the contralateral ventricle and damage the choroid plexus, causing severe complications.

**FIGURE 5:**
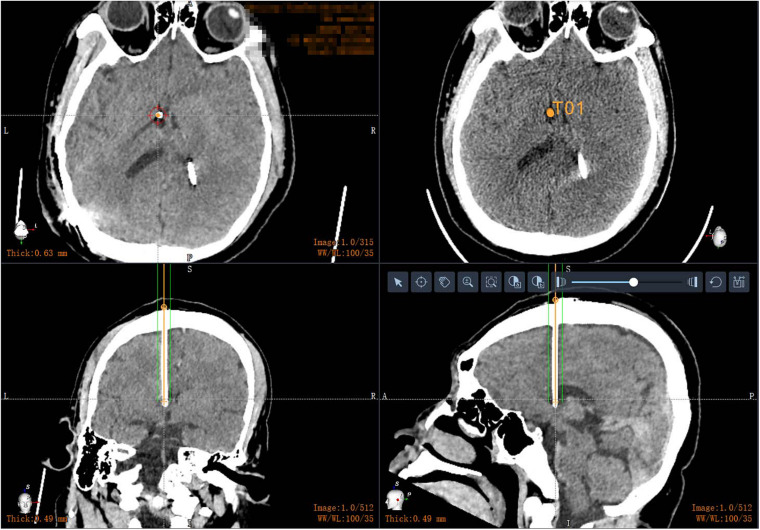
Postoperative CT in patients with small ventricles: robot-assisted drainage tube precision implantation.

By the way, robot-assisted implantation surgery offers unique adaptability to patients with small cerebral ventricles because of its accuracy and stability. A common feature of idiopathic intracranial hypertension, shunt-dependent syndrome, and slit ventricular syndrome is that the ventricular system does not enlarge and may be smaller than average, but intracranial pressure is increased (Kim et al., 2002; Bateman, 2013; Thurtell and Kawasaki, 2021). The key to treatment is to rebuild the cerebrospinal fluid circulation pathway. At present, most neurosurgeons choose to treat such conditions with a lumbar cisternal-abdominal shunt. Although this operation can relieve the symptoms of intracranial hypertension, long-term follow-up has found that some patients may develop complications such as chronic subtonsillar hernia in the later stages. The advantages of neurosurgery robot-assisted stereotactic puncture are clear in such cases. For patients with small ventricles, traditional ventriculoperitoneal shunting has a higher intraoperative risk and is more likely to result in complications such as improper drainage tube position and multiple punctures. By contrast, the size of the ventricle does not affect the accuracy of robot-assisted implantation. During the process of implantation, the drainage tube should only be implanted once, avoiding the neurovasculature and choroid plexus (Figure 5). To our knowledge, no research has been published on the correlation between the size of the ventricle and the number of punctures. However, robotic intervention means that the number of punctures is not affected by the volume of the ventricle.

With continuous advances in medical technology, clinicians have begun to use neuroendoscopy and neuronavigation to assist descending lateral ventricular shunt placement. However, a multicenter randomized trial found that endoscopic insertion of the initial VPS did not reduce the incidence of shunt failure (Kestle et al., 2003). Another prospective multicenter study found that neuronavigation in shunt surgery reduces the incidence of poor shunt placement, resulting in a significant decrease in the early shunt revision rate. However, neuronavigation is not widely used in clinical practice due to its high cost and complicated operation (Hayhurst et al., 2010).

This study has some limitations. First, the number of patients that underwent robot-assisted implantation was small, and the sample size needs to be expanded in the future. Second, this study was not a randomized trial. In future studies, patients should be randomly assigned.

## Conclusion

In summary, robot-assisted implantation is accurate, simple to operate, and practical and involves a short operation time, less trauma to the patient, and fewer postoperative complications related to surgery. In addition, the cortical puncture point and puncture channel can be adjusted according to the head CT scan of the patient, which effectively improves the success rate of puncture. In short, this technique can be widely used to improve clinical practice.

## Data Availability Statement

The raw data supporting the conclusions of this article will be made available by the authors, without undue reservation.

## Ethics Statement

The studies involving human participants were reviewed and approved by the Ethics Committee of Beijing Tiantan Hospital (Grant No. QX201600-706). The patients/participants provided their written informed consent to participate in this study. Written informed consent was obtained from the individual(s) for the publication of any potentially identifiable images or data included in this article.

## Author Contributions

All authors listed have made a substantial, direct and intellectual contribution to the work, and approved it for publication.

## Conflict of Interest

The authors declare that the research was conducted in the absence of any commercial or financial relationships that could be construed as a potential conflict of interest.

## Publisher’s Note

All claims expressed in this article are solely those of the authors and do not necessarily represent those of their affiliated organizations, or those of the publisher, the editors and the reviewers. Any product that may be evaluated in this article, or claim that may be made by its manufacturer, is not guaranteed or endorsed by the publisher.
